# Sustained Flow: Affective Obsession in Second Language Learning

**DOI:** 10.3389/fpsyg.2019.02963

**Published:** 2020-01-22

**Authors:** Zana Ibrahim

**Affiliations:** English Language Department, University of Kurdistan Hewlêr, Erbil, Iraq

**Keywords:** second language motivation, positive affect, directed motivational currents, sustained flow, affective obsession

## Abstract

A *directed motivational current* (DMC) or *sustained flow* (SF; Ibrahim and Al-Hoorie, 2019) is a motivational phenomenon characterized by intensity of engagement and sustainability of effort in which individuals display highly motivated goal-governed behavior and achieve outcomes exceeding expectations set at the outset (Dörnyei et al., 2014, 2015). This paper presents an empirical investigation into what fuels the intense and sustained motivated behavior which distinguishes the phenomenon from other types of high motivated engagement such as the ones maintained by volitional, self-regulatory measures. The qualitative (phenomenological) analysis of interview data collected from a number of subjects who had experienced SF reports two main findings. First, high motivation and intense engagement in SF are primarily the function of affective obsession with the SF experience. Once in SF, people will be mentally and affectively consumed by their experiences even at times when they are involved in other daily activities. Second, as a result of one’s affective appraisal of SF experience, one’s perception toward effort will change from viewing learning tasks as homework to perceiving engagement as one’s preferred activity conducted at one’s free time. In SF, engagement is probably considered as too emotionally satisfying and meaningful insomuch as one prefers to maintain a strong and constant sense of relatedness. As a result, effort loses its traditional connotation and therefore self-regulatory measures become unnecessary; hereby one might invest the maximum amount of effort toward learning. Theoretical implications of these two main findings are then discussed in relation to the motivational power of positive affect in directing second language learning behavior.

## Introduction

Positive psychology is concerned with valued subjective experiences such as well-being, contentment, hope, satisfaction, flow, and happiness (Seligman and Csikszentmihalyi, 2000). Among the elements that positive psychology aims to foster are positive emotions because they partially represent people’s subjective well-being and happiness (Kahneman, 1999; Oxford, 2016). Not all positive emotions are of equal interest, however. In the positive psychology literature, a distinction is made between pleasure and enjoyment. The former is the result of satisfying homeostatic needs while the latter is “the good feelings people experience when they break through the limits of homeostasis – when they do something that stretches them beyond what they are”; (Seligman and Csikszentmihalyi, 2000, p. 12). Positive psychology intends to promote enjoyment or a “mature form of happiness” which is the result of genuine engagement as in eudaimonia or flow (MacIntyre and Gregersen, 2012, p. 208). While pleasure may bring short-lived feelings of joy, enjoyment leads to “personal growth and long-term happiness” (Seligman and Csikszentmihalyi, 2000, p. 12).

Enjoyment was discussed by Aristotle as being of two different types: eudaimonia and hedonic enjoyment (Waterman, 1993). Whereas the latter is concerned with the gratification of basic human needs, the former is engagement in activities featuring “an intellectual focus, heightened attention, and optimal challenge” (Boudreau et al., 2018, p. 153). Eudaimonia is “an ideal in the sense of being an excellence, a perfection toward which one strives and, hence, it can give meaning and direction to one’s life” (Waterman, 1993, p. 678). It is assumed that hedonic happiness or pleasure is achieved when physical needs such as anger and sex are met, eudaimonic happiness is related to leading “quality of life derived from the development of a person’s best potentials and their application in the fulfillment of personally expressive, self-concordant goals” (Waterman et al., 2010, p. 41). From a positive psychology perspective, true happiness is not satisfying physiological needs, but engagement in activities which generate eudaimonia and wellbeing although they might seem as not so pleasant, such as a “mathematician solving equations, a composer working hard on the notes in a melody, a scholar pouring over reams of data, an exhausted marathon runner approaching the finish line” (MacIntyre and Gregersen, 2012, p. 208), forms of engagement which typically lead to *flow* experiences (Czikszentmihalyi, 1990).

Flow has also been referred to as optimal experience marked by focused concentration, full engagement, intrinsic motivation, control over task at hand, and enjoyment. Such experiences are also greatly linked to a better quality of life to such an extent that even people with disabilities or poor health conditions can use them for improved coping strategies and positive changes in personality and social relationships (Fave et al., 2011).

In translating enjoyment to experienced positive affect, it is assumed that positive emotions do not only bring about happiness, but they also motivate the individual experiencing them to display certain tendencies. According to the Broaden-and-Build Theory of Positive Emotions conceptualized by Fredrickson (2001), “experiences of positive emotions broaden people’s momentary thought–action repertoires, which in turn serves to build their enduring personal resources, ranging from physical and intellectual resources to social and psychological resources” (p. 218). Thus, experiences of positive emotions such as joy, interest, contentment, pride, and love create the urge to play, explore, develop self, share achievements, and connect to people.

Flow theory has rarely been applied in the L2 settings. In a pioneering study, Egbert (2004) conducted a field study involving 13 secondary school Spanish learners performing language tasks on seven separate occasions. The findings suggested the occurrence of flow and that, more importantly, teachers could “theoretically” facilitate flow in L2 learners through assigning tasks that lead to flow (p. 513). Recently, Dewaele and MacIntyre (unpublished) conducted a study to examine the frequency of flow and anti-flow experiences of 232 Spanish learners from around the world using an online questionnaire. They found that participants experienced more flow than anti-flow, and that flow was positively related to a higher skill in languages, higher standing among peers, and years of study. Importantly, the authors postulated that based on Fredrickson’s (2001) broaden and build theory of positive emotions, experiences of flow could have long-lasting consequences beyond the task engagement because accumulated experiences of these positive emotions build people’s enduring personal, physical, social, and psychological resources (Fredrickson, 2001).

Despite the assumed long-lasting influence of positive emotions and enjoyment, both flow and foreign language enjoyment (Li et al., 2018) have focused on engagement in single-learning tasks. Therefore, it is still unclear how enjoyment beyond the learning task might lead to motivated behavior for an extended period of time, which is typically required for second language learning (MacIntyre and Gregersen, 2012). To fill this gap, it is necessary to study enjoyment in long-term forms of engagement, especially to see if and how the long-term tendencies of positive affect including positive mood and happiness beyond task engagement might help sustained motivation.

## Materials and Methods

### Flow and Sustained Flow

To understand the relationship between positive affect and motivated behavior, it is important to study phenomena in which people experience long-term motivation and sustained or prolonged positive mood – rather than short-lived emotions only. *Sustained flow* (SF) or *directed motivational current* (DMC), that is “the occurrence of flow in a series of tasks aimed at achieving a certain outcome (for example improving proficiency in a second language)” (Ibrahim and Al-Hoorie, 2019, p. 51), might be an appropriate tool to provide the opportunity to study positive affect in both single tasks and throughout “a prolonged process of engagement in a *series* of tasks” (Dörnyei et al., 2015, p. 5, original emphasis).

Sustained flow or as originally termed, DMC, is a motivational phenomenon characterized by a state of intense engagement in a goal-governed activity for an extended period of time (Dörnyei et al., 2014; Ibrahim and Al-Hoorie, 2019). It is believed that while in SF, people exert an unprecedented amount of effort toward a specific goal through utilizing a facilitative structural pathway which directs the effort and helps in routinizing recurring tasks throughout the entire experience. Although not frequently occurring in people’s lives, SF is a strong burst of motivation which leads to outcomes beyond one’s expectations mainly as a result of steadily high motivation, uninterrupted engagement for an extended period of time, and constant refueling of energy through visualizing the final goal and upon passing each step along the way to the final objective.

Generally speaking, both flow and SF share the basic characteristic through which they are distinguished from other motivational phenomena: A motivational surge for a period of time which is characterized by directed concentration, full engagement, high interest, and goal orientation. However, flow theory is concerned with short-term tasks such as painting, reading, music, etc., or a series of short-term repeated identical episodes, while SF usually continues for a long period such as a few weeks, month, or even years.

Moreover, SF is an entirely goal-oriented motivational surge, while in flow the goal is important only to provide a clear structure. When studying a group of artists, Csikszentmihalyi (1988) noticed that “it was quite typical for an artist to lose all interest in the painting he had spent so much time and effort working on as soon as it was finished” (p. 3). SF is mainly triggered by a personalized goal or vision which also plays an important role in its sustainability. Relatedly, whereas flow is involvement in a task that is rewarding in itself (autotelic), but SF is concerned with engagement which leads to reaching a goal that is of more importance than the enjoyment stemming from just practicing the activity (Ibrahim Z.I., 2016).

Sustained flow is thought to have three main characteristics: a final goal or vision, a salient facilitative structure, and positive emotionality (Dörnyei et al., 2014, 2015). Early conceptualizations of the SF theory consider positive emotional loading as the third component or characteristic of any SF or DMC experience which refers to “the emotional loading of the vision which is at the heart of the DMC: anything which helps to approach the goal feels rewarding and takes on some of the positive affect associated with the outcome” (Dörnyei et al., 2014, p. 16). In describing the type of emotionality experienced in DMCs, Dörnyei et al. (2016) stressed on the difference between the autotelic enjoyment experienced in *flow* and the eudaimonic enjoyment of a DMC; whereas the former is concerned with the intrinsic joy of performing an activity for its experience only, “the ‘peak experiences’ in DMCs are derived through the energy expended on moving ever closer toward a long-term goal or vision,” and therefore, “each step along the way reproduces part of the joy that is linked to the overall journey in a fractal manner, regardless of whether or not the particular activity in question is, in and of itself, particularly enjoyable” (Dörnyei et al., 2016, p. 104). Eudaimonia is postulated to explain not only the nature of positive affect in SF, but also how it might contribute to the intense and sustained motivational behavior characteristic of all SF experiences (Henry et al., 2015).

However, little, if any, empirical investigation has been conducted on how positive emotionality experienced in SF might contribute to the unique nature of heightened and sustained motivation in SF; if and to what extend eudaimonic happiness is related and experienced in relation to the final SF goal/vision; and more importantly, what fuels the long-term motivated engagement of SF experiences. This study aims to investigate parts of these lines of inquiry, and more specifically these research questions:

(1) What is the dominant type of affect experienced while one is experiencing SF?
(2) What is the nature of positive affect in SF?
(3) Does positive affect have any impact on motivated behavior in SF? How?
(4) What is the possible role of positive affect in fueling long-term engagement of SF?

It is hoped that answering these questions might assist in understanding the motivational role of positive affect in not only SF cases but also other types of long-term engagement.

### Methodology

Sustained flow or DMC is a newly conceptualized phenomenon both in mainstream psychology and L2 motivation literature. As such, the initial stages of SF research intuitively concern understanding the phenomenon in its depth through investigating its main features, different aspects, and its motivational properties and sources. Accordingly, this research program adopted an exploratory approach through studying a number of SF cases to find common patterns among them. In order to obtain rich and detailed data of the SF phenomenon by a number of individuals and groups who experienced or were involved in at least one SF experience, a qualitative research methodology (Silverman, 2013) was seen as most appropriate to achieve the aims and answer the research questions. A qualitative research paradigm was seen as particularly useful to obtain thick and exhaustive descriptions of the phenomenon of interest, especially with regard to a newly conceptualized construct for which reaching empirical understanding and discovery is essential (Streubert, 2011). Additionally and with two participants, a quantitative affect measuring scale was used, and they rated their emotionality using their best prediction.

### Sampling

This paper reports results collected from a major and pioneering study on SF which constituted the Ph.D. project by the researcher at the University of Nottingham (Ibrahim Z.I., 2016). Recruitment started with asking adult learners and users of second languages (L2) both in the researcher’s country, Kurdistan, and in the United Kingdom to reflect back on their L2 experiences to identify any periods of extraordinary intense engagement. The main initial challenge was to capture actual SF experiences in participants who would not have any knowledge of the concept. Yet, in order to maintain validity and avoid the risk of leading participants to fit their narratives to what the researcher wanted, no details of what SF entailed were given to potential participants although enough information about the research was provided.

Upon collecting the initial responses, a screening procedure was conducted to assess whether the narratives were SF, SF-like, or merely high motivation cases. The criteria used to make this judgment were based on the theoretical foundations outlined by Dörnyei et al. (2014, 2015), focusing on goal-oriented experiences described as uniquely intense in comparison to one’s previous types of long-term engagement. The theoretical descriptions of what a SF entailed was the only criterion to recruit the sample for this study. Respondents whose initial L2 experience resembled what a SF was were considered to be appropriate participants for this study. Participants had to have experienced an extended period of uniquely intense and extraordinarily motivated behavior in pursuit of learning a second language (Table 1). A number of cases were rejected that did not meet the SF criteria especially if the experience seemed to be no-goal directed (such as the joy of watching movies in L2), or self or other-imposed highly motivated but unpleasant experiences (such as studying L2 for a national exam). The data presented in this research include data collected from seven participants via face-to-face and phone interviews and email correspondence.

**TABLE 1:** Background overview of the retrospective participants.

**Participant**	**Gender**	**Age when experienced SF (years)**	**Age at the time of interview (years)**	**How long their SF lasted**	**SF goal**	**L2 past and current experiences**
Suzan	Female	16–18	26	1.5–2 years	To prove to brother and uncle that she could also understand L2.	Was high school student at the time of SF experience. Was not engaged in intentional L2 experience at the time of interview.
Sahar	Female	18–20	25	About 2 years	To understand media content in L2.	Was university student majoring in English while experienced SF for learning Japanese. Was not engaged in intentional L2 experience at the time of interview.
Shirin	Female	25–27	27	About 2 years	To use L2 for research and teaching career.	Was engaged in pre-sessional English preparation course at the time of SF. Was not engaged in intentional L2 experience at the time of interview.
Umed	Male	28–33	37	About 3 years	To use L2 for work-related communication.	Was on a self-study L2 learning process at the time of SF. Was not engaged in any intentional L2 experience at the time of interview.
Ali	Male	29–31	34	About 1.5 years	To be successful at job interviews in L2.	Was on a self-study L2 learning process at the time of SF. Was not engaged in any intentional L2 experience at the time of interview
Kardo	Male	18–20	23	About 2 years	To prove that he is talented in L2 use.	Was in an L2-medium instruction university at the time of SF. Was not engaged in any intentional L2 experience at the time of interview
Louise	Female	23	26	3 months	To prepare for studying a post-graduate degree involving L2.	Was on a self-study L2 learning process aiming at winning a university scholarship. Was not engaged in any intentional L2 experience at the time of interview.

Moreover and in order to capture SF experiences as they occur, two individuals believed to be in SF were studied. As SF experiences are believed to be long-term engagement, it is necessary to incorporate a longitudinal aspect in studying them (Ruspini, 2002). The two individuals (Helen and Adam) were full-time post-graduate students studying English Studies at a British university (Table 2). They were interviewed about once a month for 8 months, and they were asked to rate their emotionality using a chart.

**TABLE 2:** Background overview of the follow-up participants.

**Participant**	**Gender**	**Age at time of data collection (years)**	**How long into SF**	**SF goal**	**L2 past and current experiences**
Helen	Female	27	10 months	To upgrade teaching skills in “meaningful” field of choice.	Was engaged in full-time postgraduate studies at the time of experiencing SF and at the time of interviews.
Adam	Male	31	2.5 years	To acquire research skills to better pursue an academic career.	Was engaged in full-time postgraduate studies at the time of experiencing SF and at the time of interviews.

### Data Collection and Analysis

In-depth structured and unstructured interview, seen as useful to study motivational experiences (Gillham, 2000; Silverman, 2013), was utilized to collect most of the data presented in this research. Initially, the interviewees were asked to describe and narrate their experiences in their own choosing. The respondents were asked to describe their L2 experiences during the intense motivational engagement and how their overall feelings were like throughout. The participants whose cases were deemed SF were invited to take part in the second or third rounds of interviews and asked more specific questions such as what triggered their motivational experience, what were their goals, how they studied on a daily basis, how they felt about their experience and throughout their engagement in single learning tasks and throughout the period, and why. As the participants did not have a conceptual basis to embed their experiences in, the use of unstructured interviews was viewed as particularly useful in enabling the participants to discuss “experienced but not necessarily previously reflected-on thought processes” (Henry, 2011, p. 244). A number of participants, who were not available for direct or interactive interviews, were given the option to provide a written account or protocol describing their experience in detail (Finlay, 2011).

For the two longitudinal cases, charts were also used to enable them to draw their affective states using a quantitative scale. The charts were used to measure their emotionality for a 2-week period and while in a typical single study session. Over the 2-week period, they were both interviewed three times each and asked questions about how they felt each day along with a number of other questions about their general feelings. Moreover, in each of the interview sessions, they were asked to chart their affective state and rate their emotionality from (−100 to 100) with −100 being the highest in negative emotionality and 100 the highest in positive emotionality.

Upon transcribing all the interviews and incorporating the written accounts, a phenomenological approach was deployed for the data analysis in order to capture the “universal essence” and meaning of the phenomenon in question as lived and experienced by the participants (Creswell, 2007, p. 58). This method was seen as particularly useful in understanding SF as a new phenomenon and with regard to what the participants experienced, and how and why they experienced them (Moustakas, 1994). A modified (a combination of descriptive and hermeneutic-interpretive) approach as described by Colaizzi (1978) and Moustakas (1994) was used as below:

(1) First, all the interviews and written accounts were merged together and both individual transcriptions and the whole data-set were read several times to obtain a general feel for it.
(2) Then, the researcher went through the entire data-set and extracted a non-overlapping list of the significant phrases and statements, treating them as of equal worth.
(3) Formulated meanings, that is, interpretive meanings, were produced for each statement.
(4) Clusters of themes (Colaizzi, 1978) were developed from the combination of similar formulated meanings. In developing clusters of themes, the researcher made efforts to “stay as true to the phenomenon as possible” and to “bracket” his presuppositions about the phenomenon (Hycner, 1985, p. 287, 281).
(5) Independent themes were produced from similar theme clusters (for more detail on this approach see Sanders, 2003).
(6) In parallel to this process of developing the raw data into common patterns and themes, a descriptive account was produced for each participant’s narrative reflecting “what” happened (i.e., textual description) and “how” the experience happened (i.e., structural description). Both of these descriptions were combined in a single composite description reflecting the “essence” of the experience (Creswell, 2007, p. 60).
(7) The final stage of the analysis included elements of interpretive phenomenology also in order to gain a joint understanding from what the participants and the researcher make of the experienced phenomenon (Wojnar and Swanson, 2007). The researcher reflected on the descriptive analysis “trying to make sense of the participants trying to make sense of their world,” guided by these two questions: “what is the person trying to achieve here?,” and “Do I have a sense of something going on here that maybe the participants themselves are less aware of?” (Smith and Osborn, 2003, p. 53). As with most qualitative research, however, the role of flexibility (Malterud, 2001) needs to be acknowledged here. The researcher’s presumptive conceptualization of SF might have had an impact in the overall interpretation and hence analysis of the data.

The entire data analysis was an iterative process, and the theme clusters and themes were worked through and re-examined numerous times to achieve scrutiny and scientific rigor (Fereday and Muir-Cochrane, 2006; Finlay, 2012). Close attention was paid to ensure that the final themes represent what was dominant in the data, so the final presentation describes the bulk of the data rather than deliberately selecting extracts to support specific claims (Joffe, 2004).

This research utilized a number of strategies to achieve validity. In addition to descriptive accounts as narrated by the participants, the analysis deployed a hermeneutic approach which produced scrutinized interpretations of the experiences rather than relying extensively on the participants’ perceived reasons for their experiences. Moreover, in consonance with the phenomenological approach’s “validity check” measures (Hycner, 1985, p. 291), descriptive written summaries (i.e., vignettes) were produced for each participant’s overall narrative. These summaries and a number of emergent themes were then given to a number of available participants to check whether they reflected an accurate account of their experiences.

This study was approved by the English Department’s Ethics Committee at the University of Nottingham. All the participants filled out and signed a written and informed research consent form indicating the purpose of the study, that answering to each question was voluntary, and they could withdraw from the interview at any time if they wished. To protect the identity of the participants, all the names used here in reporting and presenting the results are pseudonyms (to see a more detailed account of the methodology used, see Ibrahim Z.I., 2016).

## Results

The data collected from all the *nine* participants were analyzed deploying a phenomenological approach which resulted in *five* themes and a number of subthemes. In the data analysis process and producing themes, rather than generating statements that explicitly expressed an emotion or affective state, attempts were made to develop themes that could best reflect the data and hence explain why the participants experienced the emotionality they reported.

### Theme 1: Positive Affect

Throughout all the codes and formulated meanings related to emotionality in the analysis, positive affect emerged as the most dominant theme and was reported by all the participants. The participants used a variety of terms to describe their feelings while in their SF experiences such as “happy,” “enjoyable,” and “excited,” and their entire experience as “good,” “positive,” “pleasurable and also enjoyable,” “amazing,” and “interesting.” Among the statements used to describe their affect was “hard to describe” because it was “more than happiness” (Suzan, Q1, 2), and “a paradise-living feeling” (Kardo, Q1). Three main types of positive affect were identified as common among all or most of the participants, as below.

#### Enjoyment/Happiness

Enjoyment was reported by all nine participants. The analysis identified enjoyment at two main levels: the overall SF experience (including the mood level) and during engagement in single tasks (emotions level).

#### Overall Experience Level

The participants described the overall SF periods as enjoyable experiences. Helen, for example, thought that “*life is in general good*” (Q1), and Adam rated his overall experience as “*generally positive.*” (Q1)

A number of reasons were identified to be contributing to this feeling of positivity and enjoyment. To Adam, the source of his enjoyment was that he was making meaningful progress toward approaching his goal: “*I am achieving goals now toward the end of the second year, finished some projects, starting new projects, new developments. So it is exciting day by day*.” (Q2)

In addition to making progress toward a valuable goal, overall happiness was due to an altered perception in regard to three main aspects: altered perception toward the L2, toward oneself, and toward life in general. Shirin felt happy that she had a changed view toward the L2 while in her SF: “*It also created self-confidence in me that what appeared to be so difficult and challenging, I was about to achieve it almost easily*.” (Shirin, Q1)

Ali had acquired a new perception about his personal ability as a result of his SF and that led to happiness and further motivational tendency: “*So to be honest, this feeling of being capable made me push even further, to continue learning on a daily basis, and at the same time feel great about it*.” (Ali, Q1). Moreover and as a result of this transformation in perception about L2 learning and their abilities, the participants felt happy about the changes occurring in their entire life, as Ali further explained:

*When people were talking about the need for growth and development, I actually felt that growth in that one or one and a half years period. I could see that development in myself. I could see that on a daily basis* …. (Ali, Q2)

As can be seen from these extracts above, these changes in perception had led to changes in the participants’ motivational tendencies. Due to experiencing a sense of change in his abilities and his life after SF, Ali for example felt more encouraged to expend more effort and “*feel great about it*”; he seemed to have had an increased tendency toward continued engagement.

To many of them, the SF experience led to not only more hopes in their life and future, but also a sense of direction and meaning. Adam for example described the SF he was in as an “*eye-opening*” experience and even a mechanism whereby his life questions made more sense and acquired a sense of purpose which made his quality of life better. Therefore, he thought he was to choose this engagement regardless of his financial status: “…*So even if I was so rich that I didn’t have to work, I’d still do this*.” (Adam, Q3)

#### Task Level

At the single task level, the analysis revealed that enjoyment was the dominant type of affect as all the participants reported experiencing it most of the time while engaged in single SF tasks. For Helen, regardless of her intrinsic interest in the material, she found engagement in single tasks enjoyable because it would lead to her learning goal. As a result, even challenging tasks were experienced as interesting without causing boredom:

*It is all interesting because it contributes to the end, of the end goal, even if it is something that is really dry like for example I am not a big fan of statistics, it is a massive challenge for me, but because it contributes to the end goal*… *So there is still the interest there.* (Helen, Q2)

However, intrinsic interest in the L2 tasks, however, was reported by four participants: Suzan, Sahar, Umed, and Louise. Suzan had a pre-existing interest in watching TV shows in English prior to her SF; however, once in her SF, she changed her habit from watching TV randomly and for pleasure into watching specific shows with specific focus on learning certain aspects of the L2. She differed in how she engaged in watching TV; previously, it did not matter if she had missed a show or what type of TV program she watched, whereas while in SF she ensured she watched the episodes that improved her communicative skills, and she watched the same episodes twice – motivated primarily by the goal of learning versus intrinsic enjoyment.

Sahar also discussed how engagement in L2 tasks was perceived as interesting and fun time. Yet, it seemed that this was in part the result of her intrinsic interest in the Japanese language and culture. Therefore, she found engagement as “*interesting past time*” and a “*hobby*” initially, but later and as she encountered more challenging parts of the L2, her engagement became more goal-driven: “*it was kind of as a hobby and a past time but at the same time improving myself so, it was it was an interesting time for me yeah.*” (Sahar, Q1)

Similarly, Louise’s SF was based on learning Old Norse mainly because she was interested in reading literature produced in that language. Therefore, engagement was both intrinsically pleasurable and learning as the same time as learning to understand poetry in Old Norse was both a mechanism and a goal:

*I think it was completely hedonistic aesthetic pleasure like when you understand some part of a poetry like*… *much better than the translation, and you think about those bright images that are about it and you feel happy because you are a part of it, because this is a part of your life.* (Louise, Q1)

Some participants experienced repeated episodes of *flow* (see below). For all the participants, engagement in SF activities seemed to be one of the most, or the most, enjoyable undertaking compared to other daily involvements. For many of them, engagement in L2 learning was the first priority, not necessarily in terms of urgency, but mainly in how they affectively rated the L2 engagement, as evident from this excerpt from Helen:

Interviewer: *Would you not rather think about something else other than your study?*

Helen: *What else is there to think about? Right now that interests me nothing really*…. (Helen, Q3)

#### Satisfaction

In addition to enjoyment and happiness, the majority of the participants referred to a strong feeling of satisfaction while in their SF. Being satisfied emerged as a dominant emotional pattern reported mainly to describe the whole experience as evident from this example by Suzan:

[T]*his two years was from the happiest days in whole my life actually, I am, when I think about it, I think I relate it to satisfaction because I am satisfied with what I am doing and succeeding in what I am doing*…. (Suzan, Q3)

Contentment was not specific to the time the participants were engaged in L2 learning activities nor because of learning an L2 only. This is evident from this extract by Kardo:

*Honestly, I was listening to English podcasts 5–8 hours a day. It looks a bit of hyperbole, but this is a reality. Beside this, I was reading interesting novels, articles surfing on YouTube watching motivational and inspirational videos made me on cloud nine and I was enjoying every second of my life that time.* (Kardo, Q2)

From Kardo’s description, it is clear that in addition to spending time on learning specific L2 skills, Kardo also watched “*motivational and inspirational videos*” which did not seem to be part of the linguistic aspects of the L2 learning, but perhaps one way of making sense of his unexpected, “*crazy*” transformation which seemed “*hyperbole*” to him.

### Theme 2: Emotional Fluctuations

Although enjoyment seemed to be the dominant affective state for all the participants while in their SF, affect was not unchanging throughout. The majority of the participants referred to some instances of fluctuations in their emotionality. Since the emotionality charts were particularly utilized to collect data on these fluctuations, I report the results through the charts and in regard to fluctuations in moods (several days) and in daily emotional fluctuations (single sessions).

#### Mood-Level Fluctuations

Helen described the whole SF period (her MA studies) as being positive with regular fluctuations, but little negative affect: “… [T]*here are some periods of my time here that were definitely more happy than some others*…. *But it doesn’t go too far from that mean of happiness, you know what I mean?*” (Helen, Q4)

**FIGURE 1:**
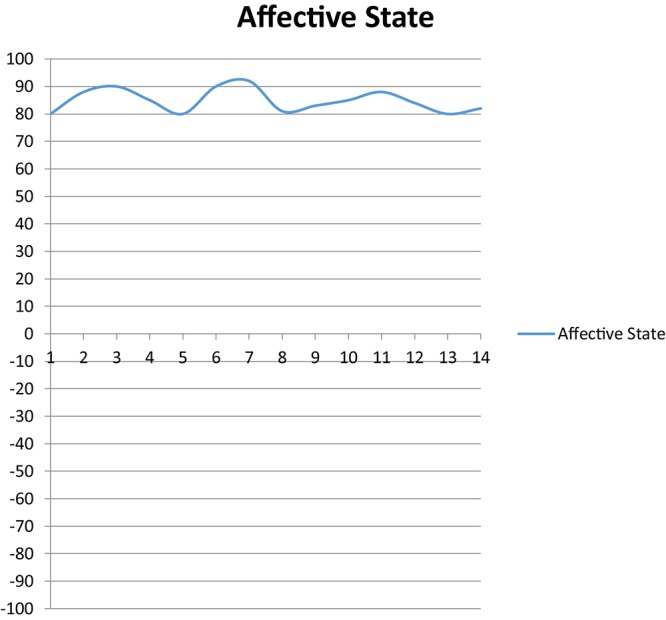
Helen’s general affective state over 14 days.

At the time of interview (three interviews within 2 weeks), despite variations, she was maintaining positive emotionality: “*Some days are better, some days are a little bit more difficult, but overall they are still pretty much constant*….” (Helen, Q5). As such, she rated her emotionality level on the developmental chart as around +80 to over +90 on average which suggested exceptionally high levels of positive emotionality (Figure 1). One possible reason for this rating was that she was engaged in autonomous writing tasks that allowed for making tangible progress.

**FIGURE 2:**
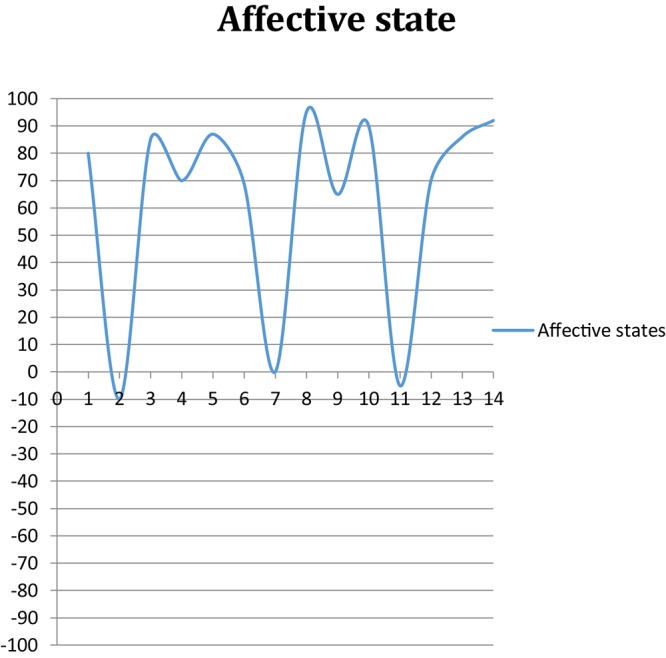
Adam’s general affective state over 14 days.

Although Adam seemed to be experiencing a somewhat similar kind of trend, he differed from Helen in how he drew his emotionality on the chart. In general, he felt he was experiencing “*excitement*” on a daily basis as he was making progress in his studies as discussed above (Q2). Nevertheless, having neutral or low-mood days was a consistent, although not frequent, pattern in his experience. When I asked him if he had any low-mood days, he described how he felt bored when he needed to spend many hours in collecting data for his studies, especially when data collection spanned extended periods or if it involved hours of waiting. Adam perceived data collection as not “*academic*” progress, but a “*technicality*” chore he needed to do, and based on this judgment he rated 3 days in the 2-weeks period as neutral or low in mood (Figure 2).

For him, judging on whether he had a positive or negative day depended on his perceived achievement in measurable terms: “*Yesterday, Sunday, was 5.25 hours. So I give myself 90% of satisfaction, because I feel I achieved something.*” (Adam, Q4). When he was studying for <3 h, he would feel less positive compared to 3 h or more.

#### Emotion-Level Fluctuations

**FIGURE 3:**
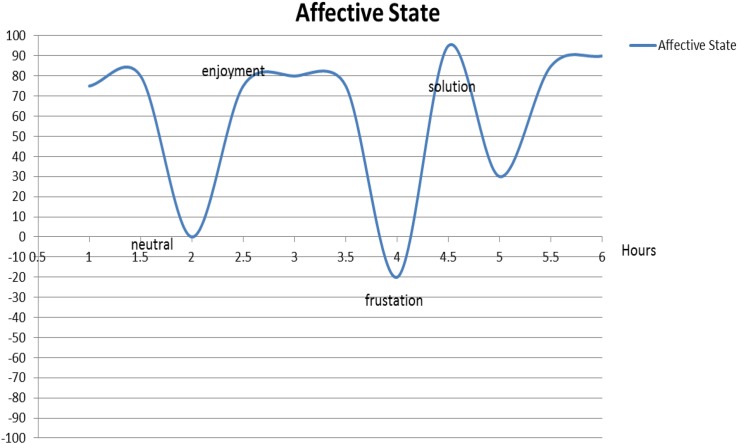
Helen’s typical affective state in a single learning session.

For Helen, enjoyment was usually the norm while engaged in a typical study session. However, moments of negative emotionality did occur. She drew on the emotionality chart that she would feel frustrated when she faced a challenge only to be followed by a sudden hike in positive affect once she found a solution (Figure 3).

**FIGURE 4:**
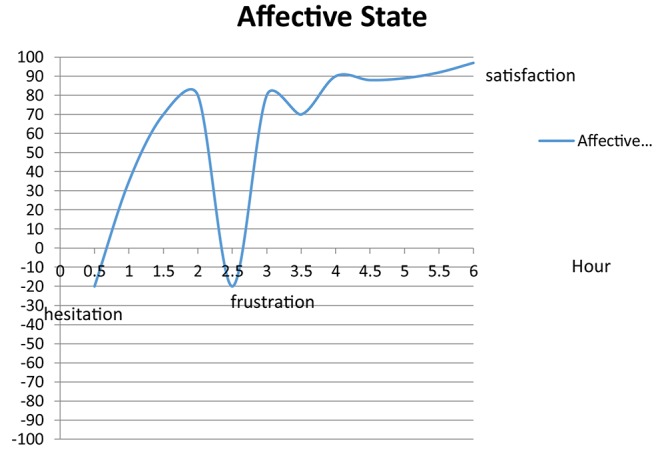
Adam’s typical affective state in a single session.

Adam stated that he would usually spend around seven consecutive (with short breaks in between) hours a day on learning. In drawing his single-session emotionality chart (Figure 4), Adam rated his sessions to be mostly positive with instances of frustration in between especially when he would not make progress due to, for example, not finding the right sources of a citation.

Upon an initial hesitation, Adam would engage in intense study sessions. As a result, not only would his sessions end with a sense of satisfaction, but he would also experience a sense of achievement and productivity: “*I have high productivity, reasonably high*… *several hours, five, six hours a day*… *This is pure productivity, not standing time*.” (Adam, Q5)

### Theme 3: Unremitting, Subdued Happiness

In general, the positive affect mostly experienced while in a SF was qualitatively different from the common usage of happiness. As described above, satisfaction was a main theme in the dataset and although the participants experienced momentary positive emotions of joy and excitement, the dominant form of happiness was rather in reference to positive mood or happiness all or most of the time – not while engaged in a learning session only. As such, a deeper form of happiness was experienced that went beyond instances of short-lived emotions.

Although Helen reported positive affect especially as a result of envisioning her final goal, it was mainly constant satisfaction rather than momentary excitement:

*It is not excitement, it is satisfaction, it is there always at the back of my head, but it is not something that I will consciously go and dig around my brain to go and find it, you know. It is there at the back of my head, I know it is there, it is present*. (Helen, Q6)

Here, it is clear that Helen felt happy beyond the times of task engagement and due to reasons beyond simply adding words to her dissertation. As a result, she had the sense that her happiness is incomparable to the overt joy of socializing or laughter:

*[I]t is not the kind of laughter and joy kind of like you would at a barbeque or a party. But it is kind of more, how do I say it, it more, I don’t really wanna use this word, but a more subdued kind of happiness*. (Helen, Q7)

As this form of happiness seemed to be unique or probably unprecedented, the participants found it difficult to share it with others.

### Theme 4: Intense Mental/Affective Preoccupation

Perhaps as a consequence of experiencing this unusual form of happiness, the participants made attempts to make sense of their experience. To do so, the participants developed an unceasing affective connection to their SF. A holistic analysis of the data revealed that engagement – or thinking about the SF – was not always restricted to a specific time. The analysis also identified that, for most of the participants, learning did not depend on a single activity such as reading in the L2. Table 3 illustrates that the participants made use of a combination of learning resources. The participants also varied in how much time they devoted to the L2 learning on a daily basis.

**TABLE 3:** Participants’ structure and daily time spent on learning.

**Participant**	**Main learning method (SF structure)**	**How much time spent daily on learning**
Ali	Used radio and TV programs, read L2 materials, spoke to foreigners in L2, attended learning sessions	Around 3–6 h
Umed	Read and translated L2 stories and newspapers, used email correspondence in L2	Around 4–6 h
Kardo	Read, watched, and listened to L2 material, attended university course in L2, spoke to people in L2	Over 10 h
Sahar	Watched L2 anime and other L2 materials, studied with a group of friends	1–3 h
Suzan	Watched TV shows and movies in L2	Around 3–4 h
Shirin	Studied at an L2 learning center, read papers, and watched TV in L2	About 6–8 h
Louise	Used vocabulary flashcards and read poetry in L2	1–5 h

As can be noticed from Table 3, while a number of participants did spend a relatively high number of hours on their learning, for at least some of them the number was low (e.g., 1 h), and for a number of participants the average was around 2–3 h a day. Nevertheless, a holistic analysis of the dataset indicated that the number of hours spent on learning might have had little relevance to the motivational intensity of the SF.

An example on the extreme side of this table was Kardo who, on some days, spent 13 h studying English and reported his daily engagement in L2 learning as follows: “… *Describing my routine in this two years, it would be speaking in English, dreaming in English, eating in English, reading in English, watching English televisions*… *all those are a great part of my life that time.*” (Kardo, Q3)

It is clear that learning had become a considerably major, if not the most significant, part of his daily occupation. However, it is important to note that this preoccupation was not always restricted to studying the L2, but also and perhaps more importantly a mental/affective obsession. An example of such an attachment to SF experience can be seen in Kardo (see Quote 2 above). Apparently, watching “*motivational and inspirational videos*” on the Internet was not aimed at learning any aspects of the target language. Rather, it indicates his mental and emotional involvement in his SF and an expression of his excitement – as also reflected in his feeling of enjoyment in his “*every second*” of his life.

Kardo’s experience was a typical example of a SF displaying intensity of cognitive, physical, and emotional engagement. Engagement in SF behavior had become the norm and exceeded his and others’ expectations.

As for the participants who did not spend as much time on learning, their experiences were still recognized for being intense, overwhelming, and obsessive. First, it is necessary to point out that the participants who spent less time on learning were virtually too busy to devote more time to studying. Louise, for example, whose SF occurred in a single summer, was meanwhile busy with a number of other responsibilities, and was only able to engage in the L2 learning in her free time. Despite the relatively little amount of time she devoted to learning, she nevertheless thought of learning Old Norse “*all the time*” and “*even in the background*”: “*Well, to tell the truth, I was not really pushing myself. Well, when you think about something all the time*… *and I think that is what helped me to do it all the time.*” (Louise, Q2). As such, because she was mentally and emotionally preoccupied with her L2 learning, she did not perceive engagement in learning as being homework performed at and for a certain time a day.

These examples suggested that all the SF experiences were considered as intense. However, this intensity of engagement was not measured by the number of hours the participants had spent on learning, but by the degree of mental, emotional, cognitive, and physical preoccupation with their SF.

### Theme 5: Constant Tendency Toward Engagement

All the participants expressed a strong desire toward engagement in SF activities, and this tendency was an always-present mental and affective orientation. The participants reported their inclination toward engagement in large part due to the prospect of experiencing positive affect upon making meaningful progress; as a result, more engagement was interpreted as the potential for more positive affect. The analysis identified the following aspects as the mechanisms by which positive emotionality led or contributed to increased tendency toward engagement.

#### SF as the Most Preferred Engagement

The participants seemed to have developed a strong emotional link to their SF insomuch that they preferred SF engagement to other undertakings. Helen for example discussed how she was obsessed with her studies and wished to devote more time to studying, sometimes leading to physical exhaustion: “*When I am not working, I feel that I should work*… *Some days, it is not funny, it is not really not funny, but I am so tired, I am absolutely drained, but even then my brain won’t stop*.” (Helen, Q8)

Here, it seemed that Helen’s preoccupation was beyond the requirements of the task (finishing her M.A. dissertation). Moreover, she reported rejecting social events and invitations such as invitations to movies, barbeques, and picnics.

Since engagement in SF behavior was perceived as emotionally satisfying, the participants strived to continue this engagement and its resulting positive affect in two main ways. First, the participants maintained an unceasing affective link to their SF even while they were not physically engaged in learning activities (i.e., in the background) as evident from this statement by Sahar: “[I]*t was definitely something that was constantly on my mind so even during lectures I would find myself scribbling in the margins of the page like in Japanese*.” (Sahar, Q2)

The second way that was used by many participants was studying in free time. This strategy was especially used by the participants who were too occupied to integrate their SF routines to their daily lifestyle. Louise, who was too busy with other ongoing commitments, made use of her free time to study Old Norse: “*Well I had to do a lot of other things*… *I had to graduate from my home university and finish my dissertation*… *But I used my free time for that, so whenever I was alone in my room*….” (Louise, Q3)

#### Personal, Me-Time

In addition to performing SF tasks in their free time, the participants believed that engagement was in main part about oneself and therefore the most enjoyable engagement. Sahar considered learning Japanese as “*time for myself*”: “*When time allowed I think, so it was like I go to for free time and as soon as like the more important things are out the way then I could just have that time for myself and learn.*” (Q3)

In all the cases, the participants had developed a personal association with their SF. Suzan for example considered her SF time as her personal time: “*Actually it was my time. I feel that it is for me only. It is like my leisure time or free time, yeah.*” (Q4). Moreover, engagement was seen as originated from one’s own decision rather than to meet any external expectations. Consequently, positive affect resulting from perceiving engagement as about one’s personal goals and benefits seemed to have led to motivation from within, as evident from this comparison Kardo put forth: “*during my holistic learning of English, no one could oblige me to study or to get ready because I was staying ready for everything all the times*.” (Kardo, Q5)

#### Flow

Many of the participants reported repeated occurrences of flow as discussed above. As a result, in addition to the belief that engagement was necessary to make progress toward a SF goal, undertaking SF activities had sometimes become enjoyable for its own sake. Sahar for example described how engagement in L2 learning sessions was her “*me-time*” in which she would immerse in the L2:

[I]*t was kind of like my personal me-time so it would be just time for me to focus on one thing and I could just shut my door in my room and just really immerse myself in the language and try to learn it.* (Sahar, Q4)

As this seemed to have become a consistent pattern throughout her SF, engagement in the L2 task was seen not only as enjoyable, but, as Sahar said, a “*treat for myself*” and “*Japanese was something that would keep me happy even if I was down so*….” (Sahar, Q5)

Regardless of whether the task at hand was intrinsically interesting, some of the participants had developed an emotional link to the media and materials they used for their learning as discussed above. As such, intrinsic enjoyment and a sense of progress/learning seemed to have functioned jointly to make engagement emotionally rewarding. Consequently, engagement was looked forward to as exemplified in this excerpt from Suzan: “[A]*ctually I was waiting, I was holding my watch.*” (Suzan, Q5)

## Discussion

The findings of this research suggest that positive emotionality is the dominant type of affect experienced in SF. Positive affect is experienced while the participants were in SF, both in single learning episodes (positive emotions) or over extended periods (positive mood). Nonetheless, in addition to this dominance, the experience of positive affect seemed to be qualitatively different from experiencing positive emotions at most other times. In describing their SF as unique experiences, the participants in large part made this judgment in affective terms. In this regard, they particularly emphasized experiencing subdued happiness, long-lasting positive mood, and satisfaction – as opposed to merely short-lived emotions of excitement or the prospect of merely reaching a new level in learning or improving a second language.

The findings suggest that the underlying reasons behind these emotional patterns are based on two different, yet complementary appraisals: making tangible progress toward a personally valuable learning goal, and a perception of productivity, skill acquisition, personal development, and transformation. These two processes seemed to have given rise to two types of positive emotionality: *anticipatory* positive emotions (current experience of an emotion because of an event in the future; Baumgartner et al., 2008) in respect of an important future goal, and *eudaimonic happiness* in regard to a sense of self-actualization and growth (Ryan and Deci, 2001). Whereas the former led to short-lived emotions of pleasure and joy, the latter was associated with unremitting long-term positive mood and satisfaction.

Importantly, the results of this research imply that the uniquely high motivational intensity of SF is perhaps fueled by positive affect and utilizing its motivating force more than by a valuable final goal/vision or a salient structure as theorized earlier by Dörnyei et al. (2014; 2015; 2016). Motivational intensity seems to be measured by one’s affective connection to their SF rather than by how much time they devote to individual learning tasks. In this study, although the participants varied in how much time they had spent on learning, they seemed to maintain a conscious mental relationship with their SF experiences. Kardo’s spending time on “*motivational and inspirational videos*” and Ali’s discussion of his happiness and progress at gatherings with friends, for example, probably mean that one appreciates and is therefore motivated by one’s emotional appraisal of the experience rather than simply on-the-task engagement. The participants varied in how much time they had devoted to learning; yet, all the experiences were considered as uniquely intense, mainly because the intensity of engagement was perceived and interpreted in light of its status as one’s most preferred or valued engagement – evident from engagement in learning in one’s free time. As a result, the participants were preoccupied with their experiences beyond the time and contexts of engagement in SF tasks. As reported in the themes, regardless of how much time one spent on L2 learning, the SF engagement was an-always-present preoccupation. Therefore, although engagement in study sessions was not always taking the participants’ greatest time, the participants were mentally and affectively consumed by their SF even at times when they were involved in other daily activities.

This preoccupation seemed vital for supporting the continuation of SF routines and also for altering the perception of effort. Relatedly, studying in one’s free time was a dominant practice among the participants suggesting that engagement in SF is not seen as homework: Engagement is probably too emotionally satisfying and meaningful insomuch as one prefers to maintain a strong and constant sense of *relatedness*. In SF, the perception of engagement changes from *a task one needs to do* to *a task one wishes to do* (Waterman, 2005). As a result, in SF, effort loses its traditional connotation and therefore self-regulatory measures become unnecessary; hereby one might invest the maximum amount of effort toward learning.

The findings provide some evidence for the impact of positive emotionality on motivated behavior. Whereas this impact has previously been assumed to be the function of both envisioning a learning goal and eudaimonia (Dörnyei et al., 2016; Ibrahim Z., 2016), the current findings suggest that the role of eudaimonic happiness might be more central than previously thought in engendering a long-lasting motivational tendency toward engagement. Therefore, the recently proposed term *SF* (Ibrahim and Al-Hoorie, 2019) in place of the original term *DMCs* might be more appropriate in describing the SF experience as it amplifies the role of positive emotionality in such experiences.

Nonetheless, eudaimonia can perhaps explain why people in SF experience the type of happiness related to positive emotions but more so to long-lasting positive mood. Yet, it is unclear if it is also behind the affective obsession people in SF usually experience which this study has found to be the primary force of motivated behavior and the alteration of effort. From the positive psychology perspective, this new conceptualization of SF as *intense motivational forms of engagement fueled primarily by positive affective obsession* has significant implications in at least two respects. First, while experiencing anticipated and anticipatory emotions (MacIntyre and Gregersen, 2012) can broaden people’s momentary and enduring resources (Fredrickson, 2001, 2009), long-lasting positive affect is perhaps more important in sustaining motivated behavior. Secondly, positive mood and positive affection beyond the immediate task engagement might be more or as paramount in inducing long-term positivity, subjective wellbeing, and happiness. More research into SF experiences is obviously necessary to further investigate these two matters.

It is noteworthy to mention a major limitation of this research. Due to the fact that the majority of the participants provided accounts of their experiences in retrospect and mostly after a number of years from the experience, it is likely that they had been biased in describing their experiences especially their emotions. That is, they were probably under the effect of remembering self rather than the experiencing self (Kahneman, 2011). Hence, they were at least partially influenced by how their experiences ended and how they recalled its details at the time of this research. Moreover, the two participants who were studied longitudinally were engaged in full-time postgraduate studies in L2 studies rather than in L2 itself. Although it has been proposed that SF is barely domain-specific, more research in participants who are engaged in L2 learning will be superior.

## Conclusion

In conclusion, the findings of this research imply that while in SF, people might approach engagement in affective terms. That is, they are motivated by the prospect of enjoyment from personal growth and eudaimonia. Therefore, we might assume that SF is primarily an affective experience fueled by long-term enjoyment and subdued happiness. Consequently, engagement in learning activities is satisfying in part because of a sense of positive change and personal development. Little is thus necessary to constantly activate self-regulatory strategies. This new conceptualization of SF has important implications for long-term motivation and the role of enjoyment in sustaining motivated behavior. In the growing field of positive psychology within second language learning, SF can have a unique contribution in increasing our understanding of the role of positive affect and enjoyment in L2 learning (Dewaele et al., 2019).

## Data Availability Statement

The datasets generated for this study are available on request to the corresponding author.

## Ethics Statement

The studies involving human participants were reviewed and approved by the University of Nottingham – English Department. The patients/participants provided their written informed consent to participate in this study. Written informed consent was obtained from the individual(s) for the publication of any potentially identifiable images or data included in this manuscript.

## Author Contributions

The author confirms being the sole contributor of this work and has approved it for publication.

## Conflict of Interest

The author declares that the research was conducted in the absence of any commercial or financial relationships that could be construed as a potential conflict of interest.
